# HCV Infection among Saudi Population: High Prevalence of Genotype 4 and Increased Viral Clearance Rate

**DOI:** 10.1371/journal.pone.0029781

**Published:** 2012-01-13

**Authors:** Ahmed S. Abdel-Moneim, Mohammad S. Bamaga, Gaber M. G. Shehab, Abdel-Aziz S. A. Abu-Elsaad, Fayssal M. Farahat

**Affiliations:** 1 College of Medicine, Taif University, Al-Taif, Saudi Arabia; 2 Virology Department, Faculty of Veterinary Medicine, Beni-Suef University, Beni-Suef, Egypt; 3 Al-Hada Armed Forces Hospital, Department of Molecular Pathology, Al-Taif, Saudi Arabia; 4 Biochemistry Department, Faculty of Agriculture, Cairo University, Giza, Egypt; 5 Zoology Department, Faculty of Science, Beni-Suef University, Beni-Suef, Egypt; 6 Postgraduate Training and Research Center, Armed Forces Hospitals, Al-Taif, Saudi Arabia; 7 Public Health and Community Medicine Department, Faculty of Medicine, Menoufia University, Shebin El-Kom, Menoufia, Egypt; University of Hyderabad, India

## Abstract

HCV is a major etiological agent of liver disease with a high rate of chronic evolution. The virus possesses 6 genotypes with many subtypes. The rate of spontaneous clearance among HCV infected individuals denotes a genetic determinant factor. The current study was designed in order to estimate the rate of HCV infection and ratio of virus clearance among a group of infected patients in Saudi Arabia from 2008 to 2011. It was additionally designed to determine the genotypes of the HCV in persistently infected patients. HCV seroprevalence was conducted on a total of 15,323 individuals. Seropositive individuals were tested by Cobas AmpliPrep/Cobas TaqMan HCV assay to determine the ratio of persistently infected patients to those who showed spontaneous viral clearance. HCV genotyping on random samples from persistently infected patients were conducted based on the differences in the 5′untranslated region (5′UTR). Anti-HCV antibodies were detected in 7.3% of the totally examined sera. A high percentage of the HCV infected individuals experienced virus clearance (48.4%). HCV genotyping revealed the presence of genotypes 1 and 4, the latter represented 97.6% of the tested strains. Evidences of the widespread of the HCV genotype 4 and a high rate of HCV virus clearance were found in Saudi Arabia.

## Introduction

Hepatitis C virus (HCV) continues to be a major etiological agent of liver disease throughout the world. HCV infection is the most serious blood borne infection in the Middle East. The virus is primarily transmitted in surgical operations such as organ transplantations, blood transfusions or by injection using contaminated syringes. HCV is a RNA virus related to the genus Hepacivirus, Family *Flaviviridae* and is characterized by a high spontaneous mutation rate [1], [2]. HCV has been classified into six major genotypes [2] and dozens of subtypes [3]. Genotypes 1, 2, and 3 are common throughout North America and Europe. Genotype 4 (HCV-4) is common in the Middle East and in Africa, where it is responsible for more than 80% of HCV infections [4]. In Saudi Arabia, HCV-4 was the most prevalent genotype followed by HCV-1 whereas genotypes 2, 3, 5 and 6 were rarely reported [5], [6], [7], [8].

In general, HCV infection usually results in viral persistence [9], and up to 30% of persistently infected patients develop chronic liver disease [10]. This poses a high risk of infection to individuals who receive organs, blood or blood products from HCV-infected donors [11], [12], [13]. Chronic HCV infection may lead to serious sequelae, including liver cirrhosis and hepatocellular carcinoma [14], [15]. A strong host immune response enhances HCV clearance [16], [17]. Thus, variation in genes involved in the immune response may contribute to the ability to clear the virus. In a recent genome based study, a single nucleotide polymorphism (rs12979860) −3 kilobases upstream of the IL28B gene, which encodes the type III interferon IFN-λ3- was shown to be associated strongly with more than a twofold difference in response to HCV drug treatment [18]. Interestingly, this mutation was found in a high rate of the population from Arabian Gulf Countries [19].

The current study was designed to: estimate the seroprevalence of HCV exposure among Saudi populations, determine the HCV genotypes in a representative sample of persistently infected patients and estimate the rate of virus clearance among the HCV exposed individuals.

## Results and Discussion

**Figure 1:**
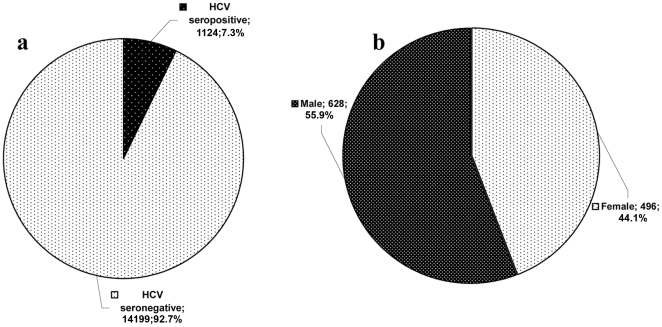
Seroprevalence of anti-HCV antibodies using chemiluminescent microparticle immunoassay. A: The total seroprevalence among the whole tested population. B. HCV seroprevalence in males in comparison to females. Results were analysed using the chi-square and the two-sided P value was 0.0034.

The HCV seroprevalence was conducted on 15,323 Saudi nationals. The overall anti-HCV antibodies were detected in 7.3% (1124/15323) of the examined individuals (Figure 1a). The HCV seropositive percentages over 4 years were ranged from (6.9–9.0%) in males and (5.3–8.5%) in females where non-significant variations were found between female and male seropositive percentages using *t* test, (P = 0.1806) (Table 1).

**Table 1:** Seroprevalence of anti-HCV antibodies using chemiluminescent microparticle immunoassay in males and females from May 2008 to May 2011.

	Male		Female		HCV Positive Percentage		
	Negative	Positive	Negative	Positive	Male	Female	Total
2008	2392	224	2251	209	8.6	8.5	8.5
2009	2569	191	2428	136	6.9	5.304	6.1
2010	1592	157	1593	109	9.0	6.404	7.7
2011	733	56	641	42	7.1	6.2	6.7
Total	7286	628	6913	496	7.9±0.5	6.6±0.7	7.3

Non-significant variation was found between female and male seropositive percentages using *t* test, (P = 0.1806).

This finding was higher than those recorded in other previous studies in Saudi Arabia [20], [21]. Between 1992 and 2002, 63,368 blood donors were screened for anti-HCV antibodies and 0.58% were found positive [20]. In another study, a higher ratio was reported −1.7% (9/528)- and the differences in the exposure rate due to ethnic origin was recorded where the anti-HCV in non-Bedouin Saudis (living in urban areas) was greater than that in Bedouin Saudis: 4.2% (7/165) and 0.5% (2/363) respectively [21]. On the other hand, Saudi patients subjected to hemodialysis showed a very high rate of HCV infection (up to 50%) with a further increase in the rate of infection among patients with end-stage renal disease [7]. In the present study, HCV prevalence of anti-HCV antibodies in males (55.9%) was found to be higher than the prevalence in females (44.1%) (Figure 1b).

HCV among acutely infected persons showed a 14–40% spontaneous recovery rate and clear viremia shortly following seroconversion while the rest of the HCV exposed individuals developed persistent infection [22], [23], [24], [25]. Chronically infected individuals possess sustained viremia usually develop persistent HCV infection. They can expect a worsening of their liver disease, potentially culminating in cirrhosis or hepatocellular carcinoma. In the current study, HCV seropositive individuals were subjected to screening for HCV RNA viral load using a Cobas AmpliPrep/Cobas TaqMan HCV assay that possesses a lower detection limit of 12 IU/ml. This assay is used for both donors and patients and has been found to be a very effective way for screening for HCV infection [26]. In some patients, the HCV-RNA level fluctuates and accordingly, HCV-RNA is undetectable. Regular follow-up of HCV-RNA is recommended at 6–12 months intervals after clearance to confirm HCV negative status [27]. Patients in the current study who became HCV-RNA negative were often re-tested 3–4 times in 12 months to confirm their clearance of the infection as recommended by Harris et al. [27]. Patients experiencing persistent HCV infection were found to comprise 51.6% (580/1124) of the total HCV exposed individuals while 48.4% (544/1124) of the HCV exposed infection cleared the infection (Table 2). There was no explanation why the infection persists in some patients and resolves spontaneously without intervention in others. Recently, host genetic variation was found to explain the heterogeneity in HCV clearance and an association between the virus clearance and the presence of a single nucleotide polymorphism (rs12979860) −3 kilobases upstream of the IL28B gene- does exist [18], [19]. The patients in that study, who showed C/C genotype, were three times more likely to clear HCV relative to those who possessed C/T and T/T genotypes [19]. The allele frequencies showed that the pattern in which the allele leading to a greater natural HCV clearance is nearly fixed throughout East Asia and also in individuals from some Arabian Gulf countries [19].Variations in genes involved in the immune response may also contribute to the ability of patients to clear the virus [19]. Conflicting results regarding the gender effect of the HCV virus clearance were recorded [28], [29]. In the current study, non-significant (P = 0.4714) differences between the ability of infected male and female patients to clear the virus (Table 2) were found. This finding agrees with those of Inoue et al. [28] but disagrees with those of Bakr et al. [29]. It is possible that interleukin 10 promoter polymorphisms (associated with rate of IL-10 production) can account for any gender disparity in HCV clearance [30].

**Table 2:** Percentage of HCV clearance among HCV infected population and comparison between percentage of clearance between male and females infected patients.

	Male		Female		Total	
	Number	Percentage	Number	Percentage	Number	Percentage
Ab+RNA−	310	49.4%	234	47.2%	544	48.4%
Ab+RNA+	318	50.6%	262	52.8%	580	51.6%
Total	628	100%	496	100%	1124	100%

Fisher's Exact Test revealed non-significant variation (P = 0.4714).

While most commercially available HCV typing assays target the 5′UTR since this region is the most highly conserved and targeted for amplification methods, it has a limited subtyping accuracy [31]. Although the preferred region for subtyping is the NS5B gene, it is not always possible to amplify this region because of primer-target mismatch. Failure in sequencing the NS5B gene has been reported by some investigators despite successive attempts to amplify cDNA with the NS5B primers [32]. It is worthwhile mentioning that all 83 samples analyzed were successfully amplified and sequenced in this study using 5′UTR.

**Figure 2:**
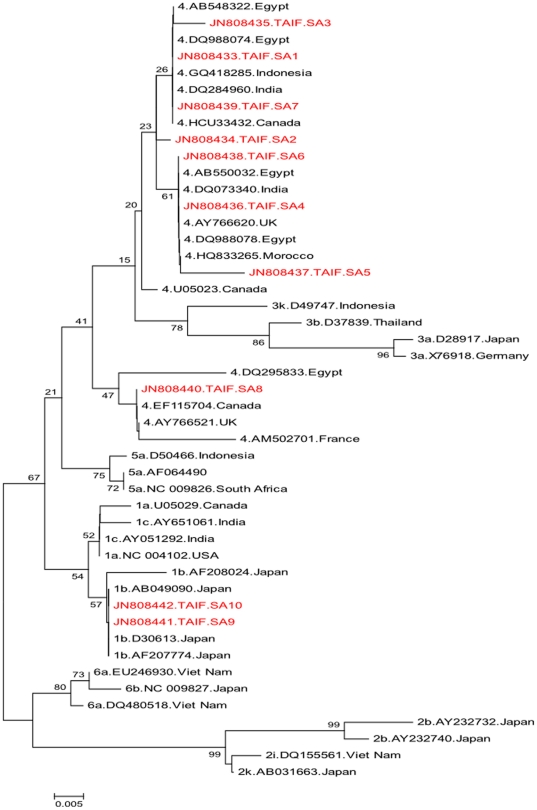
Phylogenetic analysis of partial 5′UTR sequences of HCV samples. HCV prototype sequences from GenBank were included. The evolutionary history was inferred using the Neighbor-Joining (NJ) method. Phylogenetic analysis was conducted in MEGA 4.1.

**Figure 3:**
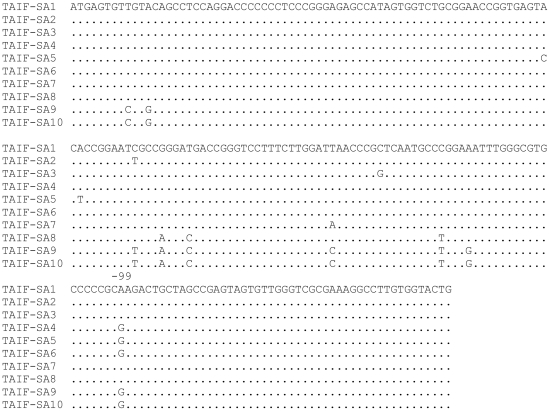
Deduced nucleotide sequence of different Saudi HCV strains based on 5′UTR sequences. Ten selected strains (TAIF.SA1-10) were included. The nucleotide sequence of (TAIF.SA1) was found identical in 75/81 of the examined strains.

Genotype 4, followed by genotypes1a and 1b, were found to be the most common genotypes in Saudi patients, whereas genotypes 2, 3, 5 and 6 were rarely found [5], [6], [7], [8]. In the current study, 81/83 strains were found to be classified as genotype 4 while only 2/83 strains were classified as genotype 1 (Figure 2). The genotype 1 strains detected in the current study possessed guanine (G) residue at position −99 (Figure 3) while 1a strains possessed adenine (A) at that residue [33], [34]. Phylogenetic analysis of the 5′UTR showed that Saudi strains (TAIF.SA9, TAIF.SA10) were identical and showed high homology to three 1b strains from Japan (AB049090, D30613 and AF207774) (Figure 2).

Among the genotype 4 strains in this study (n:81), different variants (TAIF.SA1-8) were found. The nucleotide sequence of one variant (TAIF.SA1) was found dominant in 75/81 of the examined strains (data not shown). This sequence was detected in both males and females with an age range of 14–70 years. This variant (TAIF.SA1), showed high homology to isolates: Eg3 (Accession No. DQ988074), RG309 (Accession No.DQ284960) and QC27 (Accession No. HCU33432) from Egypt, India and Canada, respectively. The remaining variants (TAIF.SA2-8) showed minor nucleotide variations. TAIF.SA3 and TAIF.SA7 were found closely related to TAIF.SA1. TAIF.SA4 and TAIF.SA6 were found to be identical and both showed high homology to TAIF.SA5 and to isolates: T63-UTR (Accession No. AB550032), 6 (Accession No. DQ073340), MOR69 (Accession No. HQ833265) and 42179 from Egypt, India, Morocco and UK, respectively. On the other hand, the TAIF.SA8 strain showed less homology to other genotype 4 strains detected in the current study but showed high nucleotide homology to isolates: cl111 (Accession No. DQ295833), QC306 (Accession No. EF115704), 14050 (Accession No. AY766521) and pt91 (Accession No. AM502701) from, Egypt, Canada, UK and France, respectively.

Therefore, current findings confirm the corrected prevalence of HCV-4 in Saudi Arabia to be at least 97.6%, i.e. greater than the previously reported prevalence observed among the Saudi population. Further, it was found that most of Saudi patients infected with a single subtype of HCV-4 however genetic variability does exist. This suggests a recent circulation of a single HCV-4 strain among Saudi patients.

## Materials and Methods

### Ethical approval

The project and data forms were approved by the Regional Research and Ethics Committee at Armed Forces Hospitals, Taif, Saudi Arabia. Written informed consent was obtained from all participants involved in our study.

### Sample collection

Plasma samples were collected at Al-Hada Armed Forces Hospital, Al-Taif, Saudi Arabia. A total of 15,323 samples were collected (7911 males and 7412 females) from individuals between the ages of 14 to 88 years during the period from May 2008 to May 2011. Samples were aliquoted and stored at −20°C till used.

### Serological assays screening for HCV

The ARCHITECT anti-HCV chemiluminescent microparticle immunoassay (version 3.0, Abbott Laboratories, Abbott Park, IL) was used for the qualitative detection of anti-HCV in plasma samples (n: 15,323) according to the manufacture's instruction. The presence or absence of anti-HCV in the specimen was determined by comparing the chemiluminescent signal in the reaction to the cutoff signal determined from a previous ARCHITECT Anti-HCV calibration. If the chemiluminescent signal in the specimen was greater than the cutoff signal, the specimen was considered reactive for anti-HCV. The reproducibility of the assay was determined by using a modified form of the EP5-A2 protocol of the Clinical and Laboratory Standards Institute [35].

### Measurements of HCV RNA

HCV-RNA was screened in seropositive samples by using COBAS Amplicor HCV 2.0 qualitative assay and the COBAS TaqMan HCV assay (Roche Diagnostics). In the COBAS Amplicor HCV 2.0 assay, the viral genome was extracted with the HCV Specimen Preparation Kit as described [36]. In the COBAS TaqMan HCV assay, the viral RNA was extracted by the automated COBAS AmpliPrep instruments and HCV RNA was amplified and detected with the COBAS TaqMan Analyzer [37].

### HCV genotyping

Randomly selected representative samples from HCV persistently infected patients (positive anti-HCV antibodies/positive HCV-RNA) were included in the study. Plasma samples from HCV real time positive patients (n:83) were subjected to HCV genotyping.

### RNA purification and reverse transcription PCR

RNA was extracted from 300 µl of plasma with 900 µl of TRIzol (Sigma), according to the manufacturer's protocols. The RNA pellet was resuspended in 50 µl of diethyl pyrocarbonate treated water (Promega) and stored at −80°C. One-step RT-PCR amplification for 5′ Untranslated Region (UTR5′) sequences was performed using one step RT PCR (Qiagen) in a total 50-µl reaction volume using 5 µl of the extracted RNA, 0.6 µM forward 5′-GAAAGCGTCTAGCCATGGCGTTAGT-3′ and reverse 5′ -CTCGCAAGCACCCTATCAGG-3′ oligonucleotides as described [38]. Samples were incubated at 50°C for 30 min, and 95°C for 15 min. DNA amplification was performed for 35 cycles each consisting of 94°C for 15 s, 50°C for 30 s, 72°C for 40 s. The last cycle was followed by a 7 min. extension step at 72°C.

### DNA purification and sequencing

Amplicons were purified and analyzed by ethidium bromide agarose gel electrophoresis. Samples showing a band of the 241 bp were excised from the gel and amplicon was purified using gel extraction kit (Qiagen Inc) for further analysis by DNA sequencing in both directions using the sense and antisense amplification primers (Macrogen Inc., Korea). All sequence data used in this study are available in the GenBank database (Accession No: JN808433-JN808442).

### Phylogenetic Analysis

BLAST analyses were initially performed to establish UTR5′ sequence identities to GenBank accessions. Comparative analyses were performed using the CLUSTAL W Multiple Sequence Alignment Program, Mega 4.1. HCV representative sequences used for the alignments were obtained from the GenBank and EMBL database. The phylogenetic trees were constructed by using the neighbour-joining method with Kimura two-parameter distances using MEGA version 4.1 [39]. The reliability of internal branches was assessed by 1000 bootstrap replications and the p-distance substitution model.

### Statistical analysis

Both Fisher's Exact Test and Chi square as well as t test were used to analyze the results using Instat software.
